# Goffin’s cockatoos use object mass but not balance cues when making object transport decisions

**DOI:** 10.1038/s41598-024-76104-7

**Published:** 2024-11-19

**Authors:** Celestine Adelmant, Antonio J. Osuna-Mascaró, Remco Folkertsma, Alice M. I. Auersperg

**Affiliations:** 1 Department of Biology, University of Oxford, Oxford, OX1 3RB UK; 2 Messerli Institute, University of Veterinary Medicine, 1210 Vienna, Austria

**Keywords:** Animal behaviour, Tropical ecology

## Abstract

Utilising weight cues can improve the efficiency of foraging behaviours by providing information on nutritional value, material strength, and tool functionality. Attending to weight cues may also facilitate the optimisation of object transport. Though some animals’ ability to *assess* weight cues has been determined, research into whether they can *apply* weight assessment during practical decision making is limited. In this study, we investigate whether Goffin’s cockatoos (*Cacatua goffiniana*) account for relative weight and unequal versus equal weight distribution when making object transport decisions, and whether sensitivity to these cues varies depending on transport mode. We conducted a series of binary choice experiments in which birds could choose to transport one of two identical, non-functional, equally rewarded objects differing only in overall weight (experiment 1) or weight balance (experiment 2) over a short distance. We found that in experiment 1, Goffin’s cockatoos preferred to transport light objects over heavy objects and seemed to rely more on weight cues to inform decisions over time, whereas in experiment 2, weight balance cues were ignored. Contrary to our predictions, Goffin’s cockatoos did not show increased preference for lighter or more balanced objects when employing higher energy transport modes (flight) compared to lower energy modes (walking). We suggest that this may be due to an insufficient difference in physical effort between transport modes due to the short distance travelled. These findings provide the first evidence of weight cues being considered to optimise object transport in birds.

## Introduction

Obtaining and using information about an object’s weight can increase the efficiency of animal foraging behaviours^1–3^. Research has shown that passerines tend to select heavier seeds for caching, which suggests nutritional assessment based on proprioceptive feedback^4,5^. Similarly, capuchins have been observed to prefer heavier stones as tools for nut cracking, indicating tool optimization^6,7^. It is likely that attention to weight cues may also play a role in making energy efficient decisions when transporting objects.

Animals manipulating and transporting resources, such as nesting materials, food, and tools, face significant costs including time, risk of predation or injury, and, most importantly, energy expenditure^8–11^. Given that object transport can consume a significant portion of an animal’s daily energy budget^12–14^, attention to weight cues may allow animals to balance the costs and benefits of object transport effectively. Such optimisation ability has the potential to improve their energy efficiency and fitness^12,15,16^.

Some animals can adopt different locomotion modes based on their immediate ecological requirements^1,17^. For example, many birds use flight to travel to, from, and between foraging patches, while walking or hopping during foraging itself^14,15,18,19^. These locomotion modes vary in energetic costs due to differences in morphology, body mass, musculature, and posture, resulting in variations in energy expenditure both between and within species^13,14,19^.

Flight is the most demanding form of locomotion in birds, particularly during take-off, ascent, and landing^20–22^. It relies on sustained, high-frequency wing flapping to generate aerodynamic lift and drag to counteract gravity, resulting in high muscular and metabolic energy expenditure^14,20–25^. In contrast, walking is less energetically demanding as the animal’s weight is supported by the ground. Metabolic demands arise primarily from muscle activity required for propulsion and balance^18,19^, making walking more energy-efficient over short distances^20^.

During both walking and flying, power output is strongly correlated with body mass. This means that carrying heavier materials significantly increases energetic costs^14,18,21^. Although no study has directly compared the energetic costs of loaded flight to loaded walking in the same individual, existing research has separately examined these costs across different species of birds^26,27^.

Studies across bird species show that both loaded walking and flight increase energy costs. In guinea fowl (*Numida meleagris*) and tufted ducks (*Aythya fuligula*), increasing load mass by up to 23% with harnesses raised metabolic costs by 17% and 16%, respectively^26,27^. Similarly, in rose-coloured starlings (*Sternus roseus*), a 7.4% increase in mass led to a 5.4% increase in energy expenditure during flight. This value was lower than expected as a result of adaptations in flight kinematics with heavier loads^17,28^.

It is not only the overall weight of an object which can impact the costs of transport, but also its weight distribution. Weight balance cues are ecologically relevant for predicting the physical behaviour of objects^29,30^. Capuchin monkeys adjust their grip on tools for balance, while Goffin’s cockatoos transport tools from the center of mass for stability^31,32^. Although metabolic costs may not significantly differ between balanced and unbalanced loads, asymmetrical weight distribution could impede flight stability by reducing speed and manoeuvrability.

According to optimal foraging theory, animals should optimise their transport-based decision making by balancing the increased costs of transporting the load with the nutritional, defensive, or clutch size benefit that the load brings^33^. They may assess these costs by utilising both the weight cues of the objects, and visual cues of their environment to assess the distance and the necessary transport mode (high up: fly; ground level: walk) to inform their decisions.

Animals use visual cues and prior knowledge to estimate object weight, but they also need to directly manipulate objects to gain proprioreceptive feedback, especially when objects look identical but differ only in weight^9,34,35^. Lambert et al.^1^ found that Goffin’s cockatoos discriminate between visually identical objects of different weights faster than primates, mastering a weight discrimination task in only 60.6 trials, compared to 331 trials for capuchins and 895.2 trials for chimpanzees^36,37^. Despite some methodological differences, this suggests that some avian species may be more sensitive to weight cues than primates.

Lambert et al.^1^ attribute differences in weight cue sensitivity between birds and primates to differences in how they transport objects and food. While primates typically walk, run or climb, carrying objects in their hands or feet, birds such as Goffin’s cockatoos transport objects by walking or flying with objects in their beaks^8^ or feet^38^. It has been suggested that the higher cost of loaded flight in birds compared to loaded walking in primates may have driven birds to evolve superior weight discrimination abilities^1^.

This study takes a novel approach to exploring the decision-making processes behind object transport in birds. Captive Goffin’s cockatoos are used as an avian model in this study due to their demonstrated weight discrimination abilities^1^ and the ecological relevance of this behaviour (they obtain much of their diet through extractive foraging, requiring the transport of large seeds and fruits to suitable perches for consumption^8,39,40^). They have also demonstrated advanced tool use skills in both wild^8,39,41^ and captive^32,42,43^ environments, including sequential tool use to access food. They have also been shown to transport tools in flight in two different studies^32,44^. Captive birds are also highly trainable, allowing us to design complex manipulations to test their cognition and behaviour in captivity.

This study aimed to determine whether birds take into account weight cues when presented with two visually identical objects with the same nutritional reward. Two binary choice experiments were conducted, in which eight subjects were presented with two kettlebell-shaped objects that differed in overall weight (heavy vs light, Experiment 1) or two dumbbell-shaped objects of the same weight but differing in weight balance (balanced vs unbalanced, Experiment 2). The subjects were then observed as they chose an object to transport to a deposit platform, either by walking across a bridge or flying.

We predicted that Goffin’s cockatoos would optimize their object carrying behaviour by: selecting lighter and more balanced objects over heavier and unbalanced ones. As objects were visually identical, we predicted that this would involve a learning process with birds choosing the lighter option more often over time. We also predicted that birds would consider weight cues more quickly when the energy requirements of transport are higher, such as when flying rather than walking.

The aim of this study is to shed light on the decision-making involved in object transport in Goffin’s cockatoos and to advance our understanding of physical cognition in Goffin’s cockatoos using a novel experimental paradigm.

## Results

### Experiment 1—overall weight

**Fig. 1:**
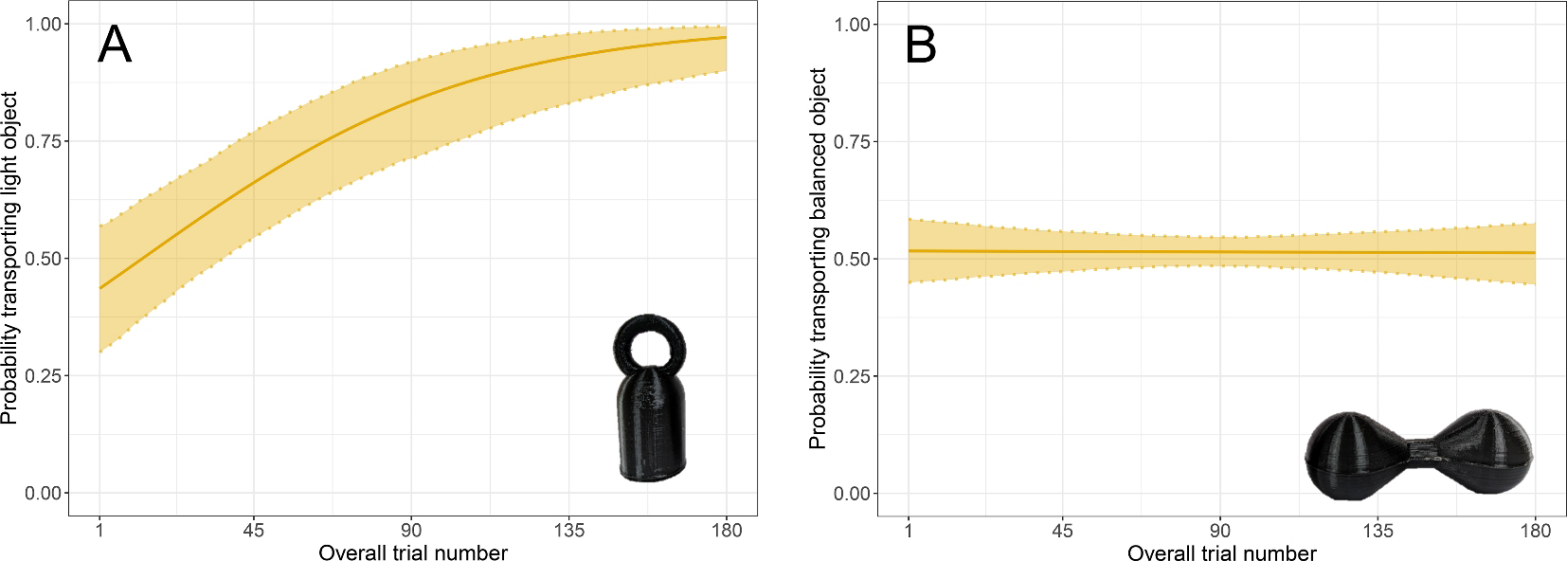
Model predictions plots: Model predictions for the probability of carrying the light or balanced object in each trial across experiment 1 (**a**) and experiment 2 (**b**). In the overall weight experiment (**a**), the probability of carrying the light object increases significantly across trials, irrespective of transport condition. In the weight balance experiment (**b**) we see no significant change in object choice across trials. The solid line indicates the fitted model, and shaded areas represent 95% confidence intervals derived from 1000 bootstraps. (**a**) Experiment 1 (Overall Weight) (**b**) Experiment 2 (Weight. Balance)

**Fig. 2:**
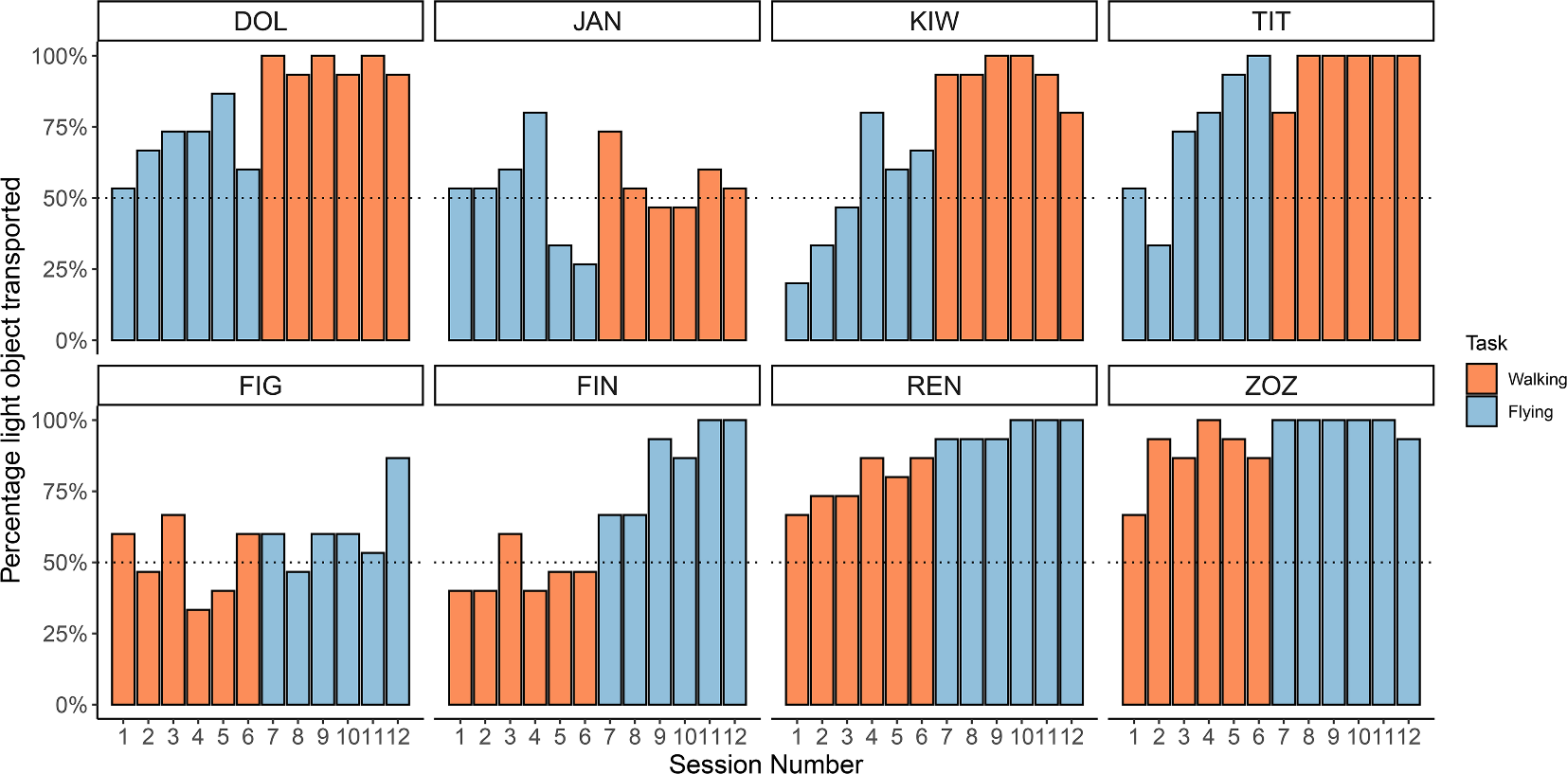
Object Weight individual raw data. The raw data for experiment 1 (Object Weight) showing individual variation in preferences and learning across sessions. Bars represent the percentage of light objects carried within each session of 15 trials for each individual separately, colour coded to show walking vs flying sessions.

Across all birds, lighter objects were transported almost three times more often than heavier objects, with light being carried in 74.4% (se±0.011%) of trials and heavy in just 25.6% (se±0.010%) of trials (Fig. 1A). Though all birds chose the light object more often than the heavy object, for two birds this preference was not significant on an individual level (Figaro–Male; 16 years, and Jane–Female; 7 years), carrying light objects in 56.1% (se±0.037%) and 53.3% (se±0.037%) of trials, respectively (Fig. 2). We further investigated these individuals and found that both birds exhibited significant side biases. Specifically, Figaro consistently chooses the object on the left (90.5% se ± 0.022%), while Jane tends to choose the object on the right (72.8% se ± 0.033%–see Supplementary for full details).

Contrary to our predictions, the full model revealed that birds did not learn to preferentially choose light objects significantly faster in flying trials than walking trials (model OW1, full-null model comparison: *χ*^2^= 4.768, *df* = 3, *p*-value = 0.190, interaction effect: *χ*^2^= 1.371, *df* = 1, *p*-value = 0.242). The reduced model revealed that over the entire experiment individuals showed a preference to carry the lighter object over time, independent of transport condition (overall trial number effect: : *χ*^2^= 9.376, *df* = 1, *p*-value = 0.002).

Switching behaviour was observed in 38.5% (se ± 0.013%) of all trials in experiment 1. All individuals showed some level of switching, with some birds switching more often than others. Individuals switched between objects between 0 and 13 times per trial, with the average number of switches ranging between 0 and 2 per trial across all individuals (see supplementary materials for further details). The full-null model comparison revealed that change in probability of birds switching across trials did not differ markedly between transport conditions (model OW2, full-null model comparison: *χ*^2^ = 6.5846, *df* = 3, *p*-value = 0.086, interaction effect: *χ*^2^= 0.417, *df* = 1, *p*-value = 0.518). The reduced model showed an increase of the probability of switching over the course of the experiment (*χ*^2^ = 4.844, *df* = 1, *p*-value = 0.028), however we caution the interpretation of this result since the full-null model comparison did not reveal significance (see supplementary materials for model outputs).

### Experiment 2—weight balance

In the weight balance experiment, balanced objects were not transported significantly more often than unbalanced objects, with balanced objects chosen in 50.9% (se ± 0.016%) of trials, and unbalanced objects chosen in 49.1% (se ± 0.016%) of trials (Fig. 1B).

Overall, birds did not carry balanced objects significantly more often when flying than walking (model WB1, full-null model comparison: *χ*^2^ = 2.234, *df* = 3, *p*-value = 0.525). Nor did subjects learn to preferentially choose balanced objects more quickly in flying trials than walking trials (interaction effect: *χ*^2^ = 0.027, *df* = 3, *p*-value = 0.869).

Switching was much less frequent in this experiment, occurring in only 15% of trials, and the average number of switches per trial per individual ranged between 0 and 0.65 (see supplementary materials for full details). Similarly, there is not a significant difference in switching frequency across the walking and flying tasks and switching frequency also does not change significantly across trials (full-null model comparison: *χ*^2^ = 5.104, *df* = 3, *p*-value = 0.164, interaction effect: *χ*^2^ = 0.692, *df* = 3, *p*-value = 0.406).

## Discussion

The results of our study strongly support the prediction that Goffin’s cockatoos will freely choose to carry lighter objects over otherwise identical heavier objects when rewards are equal, irrespective of transport mode. Additionally, our results indicate that the birds’ preference for lighter objects increases over time suggesting that they can learn to attend to weight cues to facilitate transport more over time in a repetitive task. These findings not only corroborate a previous report by Lambert et al.^1^ that Goffin’s cockatoos can discriminate between visually identical objects differing in weight, they importantly also suggest that Goffin’s cockatoos consider weight-only cues during decision making in a manner that optimizes energetic investment in a transport task. If this utilization of weight cues occurs in the wild, it may increase the efficiency of foraging behaviours as transporting lighter objects is less energetically demanding and less likely to cause injury. These findings provide the first evidence of birds using weight cues to optimize transport decisions.

Although the preference for light objects was significant when all birds were grouped, an individual analysis revealed that two of the eight birds, namely Figaro (male, 16 years old) and Jane (female, 7 years old), did not show a significant preference. This lack of preference may be due to the consolidation of an inflexible strategy such as a side bias in these individuals^32^. Further investigation has revealed that Jane and Figaro exhibit significant side biases. These individuals also showed reduced switching compared to the other birds, though this result was not statistically significant (see supplementary materials). In birds that showed a preference for lighter objects, the heavy object was still chosen up to 35% of the time. We suggest that the unnecessary transport of heavy objects may be due to a trade-off between the extra physical effort required to transport the heavy object and the cognitive effort involved in processing proprioceptive feedback during the decision making processes^32^. Indeed, delaying decision making and switching between objects can impose an energetic, cognitive, and temporal cost that may outweigh the physical cost of carrying the extra weight.

Despite the cost that switching may incur, it is rather common, appearing in 38% of trials in the overall weight experiment, with individuals switching from one object to another between one and thirteen times consecutively per trial. Birds were found to switch to lighter objects more often than to heavier objects, again corroborating previous findings that Goffin’s cockatoos more frequently switch to ‘correct’ items than to ‘incorrect’ items in decision-making tasks^1,32^. Switching behaviour suggests that individuals show uncertainty and are actively trying to gather further information about objects to make an informed decision^32^. We therefore suggest that examining switching behaviour in a similar binary task where the difference in weight between the two weight options is gradually decreased, could serve as a useful method to measure cognitive flexibility and metacognition in future studies^32,45,46^.

Contrary to our predictions, we found that birds did not choose light objects more often, nor did they learn to rely on weight cues faster, when the energetic costs of transport were higher (flying compared to walking). A possible explanation for this lack of response could be that birds do not base their decisions on the upcoming transport^47^. However, this is unlikely due to the visibility of the apparatus prior to the task, the predictability of the transport mode (transport conditions were completed in blocks), and previous evidence (a recent study on tool set transport in Goffin’s cockatoos found that the birds were able to flexibly transport either single tools or tool sets in flight depending on the requirements of the puzzle box they were needed to solve, providing evidence of flexible goal-oriented decision-making^32^).

Alternatively, it is possible that the energetic costs of flight are not much higher than those of walking in Goffin’s cockatoos. Studies in rose-coloured starlings^17,28^ and cockatiels^48^ have found minimal increases in metabolic costs of loaded flight compared to unloaded flight due to behavioural alterations in flight kinematics. In these studies, birds used behavioural responses to increase the efficiency of their flight muscles, reducing the energetic impact of increased payload mass. Thus, if the costs of loaded flight are low, loaded flight and loaded walking may incur comparable energetic costs, as walking is a slower mode of transport^20^. Due to the taxonomic and ecological differences between the species in these studies and Goffin’s cockatoos, a direct analysis of the metabolic costs of different forms of transport in Goffin’s cockatoos would be required to truly disentangle these findings.

The short, horizontal transportation distance used in this study (which was constrained by the available space inside the aviary testing room), are not likely to reflect the transport distances Goffin’s cockatoos habitually undertake in the wild. Goffin’s cockatoos forage in seasonal patches, often picking up fruits from the ground and transporting them into trees to extract the seed inside^40^. This transport involves vertical flight of up to 25 metres. In order to better understand the object transport decision making in these birds, it is important to use ecologically relevant transport distances, so future studies may choose to increase the distance of transportation and include a vertical object transport condition.

Our study revealed that the birds did not use weight balance cues at all to inform their object transport decisions. Subjects showed no significant preference between balanced and unbalanced objects. This lack of attendance to balance cues can either be due to the birds’ inability to detect these differences, or because these differences do not significantly affect their transport.

It is possible that the Goffin’s were not sensitive to the unequal weight distributions of the objects. For birds, most ecologically relevant objects such as fruits usually have relatively equal weight distributions, making it easy to visually determine the centre of mass^30^. The lack of exposure to objects with highly unequal weight distributions in nature may have limited the evolution of mechanisms to detect and respond to such cues.

Alternatively, it is possible that the object shape and the way in which the birds held them may have led to illusions of balance. Weight perception is susceptible to illusions, as, for example, smaller, more compact objects feel heavier. Even in humans, differences in surface material, temperature, and colour, can also lead to differences in perceived weight^49^. Such illusions may translate to weight balance. Naylor et al*.*^50^ posited that cognitive expectations are enough to create illusory weight differences in humans. Thus, picking up two visually and overall mass identical objects sequentially may have diluted any perceived differences between them due to the expectation created from the previous object.

It could be, however, that the birds *were* able to discriminate between the two objects based on their weight balance, but that this difference did not affect transport significantly enough to choose one over the other. Given that the transport distance was so short, it is possible that this did not impose a significant cost on the birds. Indeed, when experimental videos were coded, we recorded no clear behavioural difficulties such as head tilting, or unbalanced flight, and all transport trips were successful. To disentangle these different possibilities, we would need to investigate the birds’ ability to discriminate between objects based solely on weight balance. This could be done either by investigating if birds can be trained to reliably choose one object over another using weight cues alone^1^, or by designing a novel experiment where weight distribution differences of tools alter the functionality of tools and therefore the costs of tool use.

Goffin’s cockatoos may also be able to adjust their behaviour and grip to reduce weight asymmetry^51^. The objects were designed to prevent the birds from gripping the unbalanced object at its centre of mass (see Fig. 4). However, by gripping the object’s centre more tightly, birds may be able to reduce the perceived difference in weight between the two sides of the dumbbell. To investigate this, we propose a follow up study that investigates the differences in grip between balanced and unbalanced objects using a plasticine grip, plaster casting, and 3D reconstruction. The results from this may thus provide insight into the mechanisms that birds use to cope with unbalanced objects during flight.

The minimal amount of switching in this experiment (observed in only 15% of trials overall) may indicate that the difference in effort required to carry balanced and unbalanced objects is not significant enough to affect decision-making. Goffin’s cockatoos were also equally likely to end up with a balanced or unbalanced object after switching, suggesting that individuals in this setup rely much less on weight balance cues to make their decision as they are not trying to gather further information about the objects, nor are they using this information to inform their decisions. It is possible that the temporal and cognitive demands of deciphering which object is balanced is more cognitively demanding than carrying the unbalanced object is physically demanding.

This study presents the first evidence of transport optimisation in birds using weight cues. We found that most birds choose light objects over heavy ones when rewards are constant, suggesting that birds use object weight cues when making object transport decisions. We also found that Goffin’s cockatoos do not use weight balance cues to inform transport decisions which may be due to a lack of weight balance-sensitivity, inadequate stimulus strength in our setup, or a mechanism of overcoming the effects. Finally, we found that these Goffin’s cockatoos did not alter their choices with respect to overall weight or weight balance when using different forms of transport. We suggest that this could be attributed to lower-than-anticipated differences in the metabolic cost of carrying loads during flight compared to walking. The results from this study pave the way for future investigations into the functional and non-functional use of weight perception in birds and provide some insight into the possible explanations for increased weight sensitivity in birds.

## Methods

### Subjects, housing, and experimental history

#### Subjects and housing

We tested eight captive-born and hand-reared Goffin’s cockatoos (7 adults, M:5, F:2, and 1 sub-adult M:1). Subjects are housed together in a large, environmentally, and socially enriched aviary at the Goffin Lab in Vienna, Austria. This facility comprises both a climate controlled indoor area (45m^2^; 3 to 6 m high), and outdoor aviary (200 m^2^, 3 to 4.5 m high), with 12-hour light and dark cycles, and *ad libitum* food supply. During voluntary testing, individual subjects are called by name into an indoor testing room, where they are visually separated from the group to eliminate social learning between birds taking part in the experiment. Individuals can be identified using colour coded foot rings.

#### Experimental history

Seven out of the eight birds had previously taken part in a weight discrimination study where subjects were trained through positive reinforcement to choose either heavy or light Fimo™ clay balls (see^1^). Four out of the eight birds had also taken part in a tool transport study in which subjects transported long ‘stick’ tools to a platform (see^32^). To ensure that subjects did not retain a preference, we carried out a pre-test phase where both heavy and light items were rewarded (see supplementary materials for details).

#### Ethics

All experiments are appetitive, behavioural, and non-invasive, and subjects participated in experiments on a voluntary basis, therefore these studies are not classified as animal experiments under the Austrian animal experiments act (TVG 2012). Nevertheless, all experiments were approved by the Ethics and Animal Welfare Committee of the University of Veterinary medicine, Vienna (ETK-179/11/2022), as well as the University of Oxford, in accordance with good scientific practice guidelines and national legislation.

### Apparatus and objects

#### Apparatus

**Fig. 3:**
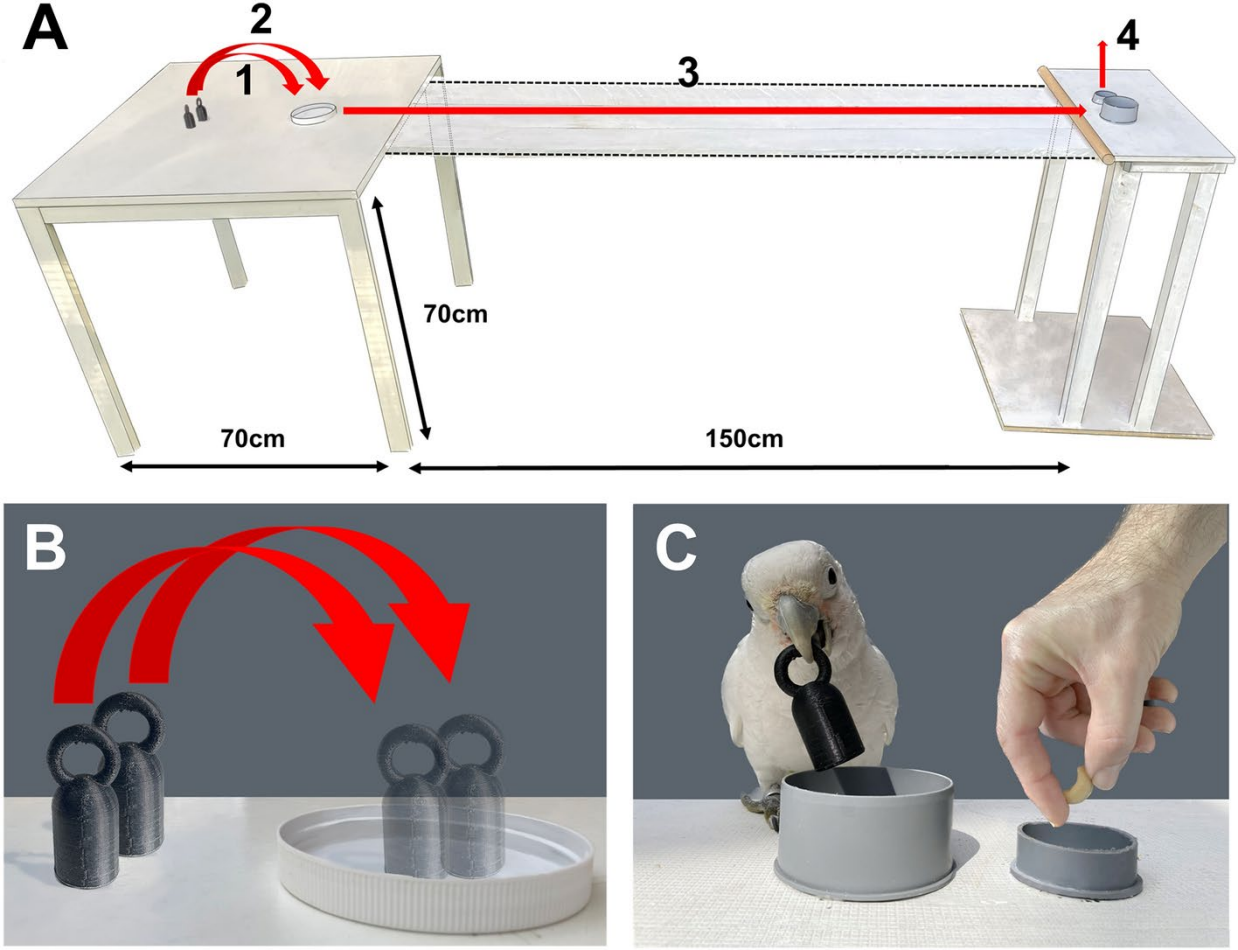
Experimental apparatus and procedure: (**A**) Table with kettlebell-shaped objects that are moved to a dish (1 and 2) for an initial proprioceptive feedback, transported from the table to an elevated platform (3) by walking across a bridge or flying if no bridge is present, and exchanged for a reward when the object is released in a dish (4); (**B**) Detail of the starting point where birds obtain proprioceptive feedback of the objects; (**C**) Detail of the exchange of the object for reward.

Two experiments were carried out that each used the same basic apparatus: a table, an elevated platform of the same height, and a removable bridge between the two (see Fig. 3). The white varnished wooden testing table (depth 70 cm, width 70 cm, height 70 cm) had a white, plastic dish (diameter 12.5 cm, height 1 cm) glued to its surface. The white wooden platform (depth 20 cm, width 50 cm, height 70 cm) had two grey, plastic dishes glued onto its surface, one dish to deposit the object (diameter 7.5 cm, height 4 cm) and another to obtain the reward (diameter 5 cm, height 2 cm). The table and platform were placed 1.5 m away from one another in the testing room. A removable white wooden bridge (length 150 cm, width 40 cm, thickness 2 cm) was placed between the two tables only during the walking condition.

#### Objects

The objects used for experiments 1 and 2 were designed using Openscad software, and 3D printed using non-toxic black Polylactic acid (PLA). Objects were designed in a way that allowed mass to be altered while objects remained visually identical.

**Fig. 4:**
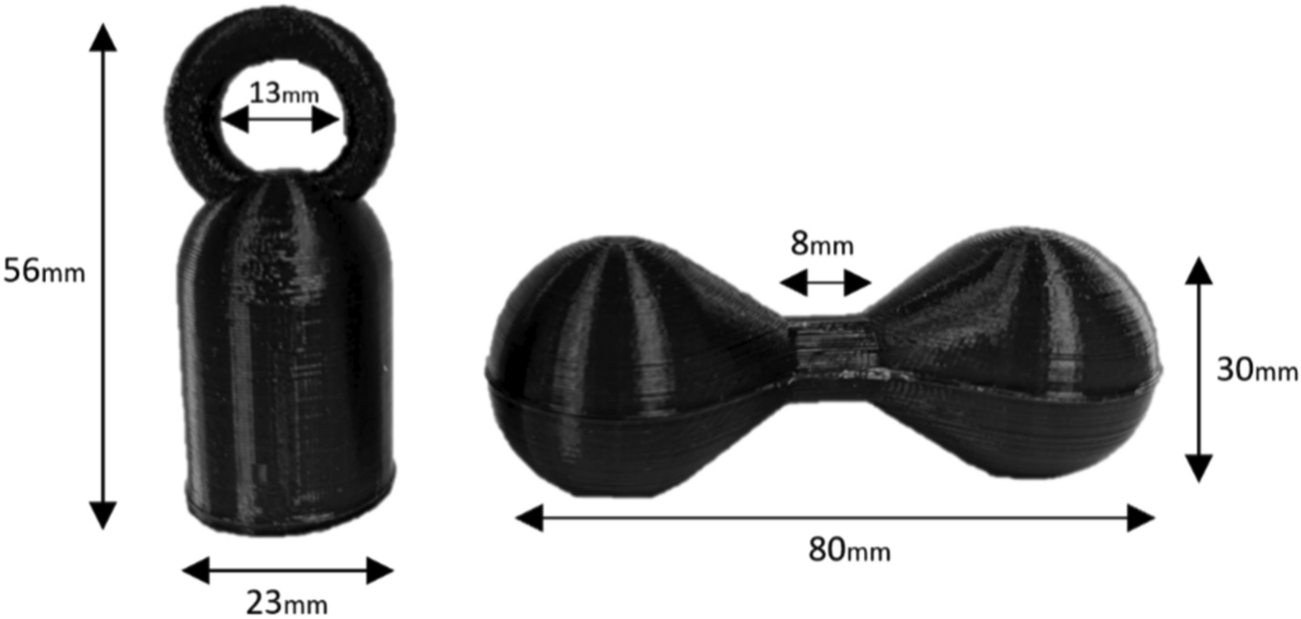
Images of objects used for experiments. Images of objects used for experiments. Experiment 1 (left) used kettle-bell type objects (56 mm tall, 23 mm diameter), with a hollow base filled with different weights to alter overall mass. Experiment 2 (right) used dumbbell-type objects (80 mm in length, 30 mm in diameter with a grip of 8 mm), with weights placed in either end to alter weight distribution. In the balanced object the centre of mass was in the handle (40 mm across) whereas in the unbalanced object the centre of mass was on the sloped edge (65 mm across) where the bird was not able to grip. The bird therefore gripped the object 25 mm away from its centre of mass.

*Experiment 1 – overall weight* The objects in the overall weight experiment (experiment 1) were kettlebell-shaped, with a hollow, cylindrical base, and a circular solid handle (see Fig. 4 for dimensions). Lead weights of different masses were fixed inside the hollow base using glue and Fimo™ clay and were sealed inside with a 3D printed lid. The centre of mass was directly below the handle. As subjects differ in age, sex, weight, and motivation, the mass that each subject considers ‘heavy’ was expected to be different. To ensure equivalent motivation between stronger and weaker birds, we carried out a pre-test procedure (see Supplementary materials for details) to determine the maximum weight that each subject is willing/able to carry. Therefore, the mass of the objects used in experiment 1 was subject-dependent (see supplementary Table S1).

*Experiment 2 – weight balance* The objects used in the weight balance experiment were dumbbell shaped, with two hollow, spherical sides and a small, solid grip handle in the centre (see Fig. 4 for dimensions). Lead weights were also added to each end of the objects to achieve balanced (weight equal on both sides) and unbalanced (weight different on each side) objects with the same overall mass. The unbalanced object was designed in such a way that its centre of mass was 65 mm across, on the slope which the birds could not grip, whereas the balanced object was designed to have a centre of mass within the handle where the birds gripped the object. As the choice in this experiment was between two objects of the same mass, strength-linked differences in motivation had a smaller impact. We therefore assigned birds to a ‘strong bird’ group (if they carried >70 g in experiment 1) and a ‘weak bird’ group (if they carried <70 g in experiment 1). The stronger birds carried dumbbells with overall weights of 60 g, while the weaker birds carried dumbbells with overall weights of 30 g (see supplementary materials Table S1 for full details).

### Procedure training

The birds were given a single session of 6 trials to learn the procedure. For this, one randomised object (heavy or light) was placed in the dish, and the bird was asked to transport it to the deposit dish for a reward. As the birds were rewarded for both heavy and light objects, this step helped to reduce any heavy or light weight-bias that may have remained from previous experiments (such as^1^).

### Experimental procedure

Experiments 1 and 2 have identical experimental procedures, with the only difference being the objects used. One object was placed in front of the subject, 10cm from the dish (see Fig. 3). The subject was asked to place this object into the dish using a pre-trained^1^ ‘give me’ command, obtaining a reward (sunflower seed). Once the object has been moved into the dish, the second object is placed in the original position, and the subject is, once again, asked to move the object to the dish. The order in which the objects were introduced (heavy/imbalanced vs light/balanced) was semi randomised for each trial (see below). This step ensures that in each trial, the bird has received proprioreceptive feedback from both weights. Once both objects were placed in the dish, the experimenter picked up both objects at the same time and placed them back down on the dish, ensuring that both weights were similarly positioned and equidistant to the platform so that the subject had the same visual experience of both objects. This involved placing objects that had fallen over upright, however the relative position of each object to the subject remained the same. The experimenter moved away to the deposit dish. The subject was then able to choose one of the two objects and transport it 1.5 m to the platform. When one of the objects was dropped into the deposit dish a high-quality reward (1/10th cashew) was immediately dropped into the reward dish.

**Fig. 5:**
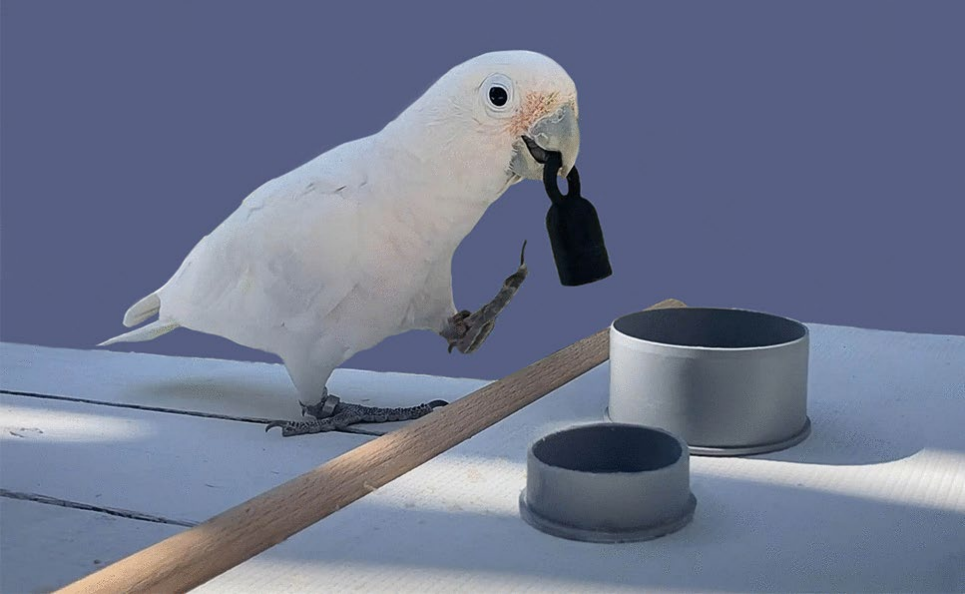
Side view of a bird depositing an object into the deposit tube before receiving a reward.

The experimenter wore reflective sunglasses, avoided head movements, and looked only at the deposit platform (not at objects) to reduce the effects of involuntary cuing and gaze following bias (Fig. 5).

For each experiment, subjects were tested in two conditions: (a) walking-transport, and (b) flying-transport (see Video S1). In the walking transport condition, the bridge was present, so the bird walked from the table to the platform, whereas in the flight transport condition, the bridge was absent, and the bird had to fly from the table to the dishes. The object the bird chose to deposit in the dish was recorded for each trial, as well as any switching between objects prior to transport. Switching was defined as the single or consecutive grabbing and releasing of one object and then the other. Switching was recorded as ‘number of switches’ from the first object picked up to the object chosen, which ranged between 1 and 13 switches per trial (video-coded post-experiment). This information was then coded to ‘binary-switch’ (whether any switching has occurred – yes/no), and ‘binary-swap’ (whether the final object chosen was different to the first object picked up).

To reduce bias, the object which was first presented and moved into the experimental dish at the very start of each trial (heavy/light or balanced/unbalanced) was semi-randomised. We generated five blocks of 15 semi-randomised ‘first object presented’ values (equal numbers of heavy and light in each block, with a maximum of three of the same objects in a row). These blocks were rotated within subjects to dictate which object is presented first in each trial. For each experiment, subjects received 6 sessions per condition (6 walking sessions and 6 flying sessions), with 15 trials per session. The order in which they received their sessions (walking sessions first or flying sessions first) was semi-randomised and kept constant for both experiments, accounting for age, sex, and strength as much as possible (see Supplementary Table S1).

### Statistical analysis

We ran a series of generalized linear mixed models (GLMMs^52^) with the statistical program R (version 4.2.3^53^, using the function ‘glmer’ of the package ‘lme4’^54^, using the optimizer “bobyqa”^55^ with 200,000 iterations. To address the question if Goffin’s cockatoos would optimize their carrying behaviour, we fitted one logistic GLMM for each experiment including data from all sessions with “lighter object” and “balanced object” as response variables, respectively. Each of these data sets consisted of 1440 observations taken from 8 individuals. We were mainly interested in learning effects over time and whether this depends on the energy requirement of transport. Thus, we included the interaction of the transport condition and trial number within transport condition as test predictors. As control predictors we included first object presented and the first transport condition the bird was exposed to, since these might affect individuals’ preference to carry a certain object. We further included the weight of the heavy object as percentage of body weight as control predictor to account for differences in motivation between birds. To account for overall learning effects, we also included overall trial number as a control predictor.

To further explore Goffin’s cockatoos’ behaviour in this experiment we additionally fitted logistic GLMMs for each experiment that included switching and swapping as response variables, as well as a Poisson GLMM including number of switches as a response. For these responses we decided to only include the observations of each individual’s first transport condition, as their behaviour in the second condition will probably be severely influenced by their experience in the first. This resulted in 720 observations from 8 individuals per data set. Similarly to the above models, these included the interaction of transport condition and trial number within transport condition as test predictors. As control predictors we included first object presented, first side pickup and the weight of the heavy object as percentage of body weight.

To avoid pseudo-replication, all models included the random intercept effect of subject identity. Additionally, to model day specific variation in motivation/mood, we included a factor combining subject and session number nested “sessionID” within subject as random intercept effect. We included all possible identifiable random slopes to avoid overconfident models and to keep the Type I error rate at the nominal level of 0.05^56,57^. We removed correlations among random slopes and random intercepts when they appeared unidentifiable (indicated as being close to +1 or −1)^58^. For all models, covariates were z-transformed to ease model convergence and achieve easier interpretable model coefficients^59^.

After fitting the models, we checked model assumptions and assessed model stability. For each model we verified absence of collinearity by calculating the Variance Inflation Factor (VIF) for the model excluding any interactions and random effects using the R package “car” version 3.0-12^60^. This revealed that collinearity was not an issue (max VIF = 1.33). We visually inspected and confirmed that the best linear unbiased predictors (BLUPs) per level of the random effects were approximately normally distributed^52^. To estimate the stability of the model, we excluded the levels of the random effects one at a time and compared the resulting estimates with those obtained from the model based on all data. This revealed that some of the models were only of moderate stability (see results tables), further inspection revealed this can be traced back to variation in the response between individuals resulting from having a sample size of 8 individuals. We checked for overdispersion in the Poisson models, which revealed that none of the responses were overdispersed (dispersion parameter OW: 0.877, WB: 0.802). To avoid an increased type 1 error risk due to multiple testing^61^ we first tested the overall effect of the test predictors. Therefore, we compared each full model with all terms included to its respective null model lacking the test predictors but otherwise being identical in the fixed and random effects using a likelihood ratio test^62^. If this revealed significance, we then continued with testing the significance of individual fixed effects by reducing model complexity (starting with dropping the interaction and continuing with a main effects only model if the interaction did not reveal significance) and comparing the simpler with the more complex model using likelihood ratio tests^62^ using the R function drop1.

We determined the 95% confidence intervals for the model estimates by applying the function ‘bootMer’ of the package ‘lme4’^54^, using 1000 parametric bootstraps.

All graphs were constructed using R version 4.3.2 (R development Core Team, 2022) and packages: ggplot2 (version 3.4.1^63^), lme4 (version 1.1-30^54^), ggbeeswarm (version 0.7.2^64^), car (version 3.1-2^60^), dplyr (version 1.1.0^65^), tidyr (version 1.3.0^66^).

## Supplementary Information

Supplementary Materials 1.

Supplementary Materials 2.
