# Supplementary material for: Goffin’s cockatoos use object mass but not balance cues when making object transport decisions
Source: Sci Rep. 2024 Nov 19;14:27265. doi: 10.1038/s41598-024-76104-7 (PMC11576884; doi:10.1038/s41598-024-76104-7)
Supplement: Supplementary file 1 — Supplementary Materials 1. [file 41598_2024_76104_MOESM1_ESM.docx]

**Supplementary Materials for:**

**Goffin’s cockatoos use object mass but not balance cues when making object transport decisions**

**Célestine Adelmant^1*+^, Antonio J. Osuna-Mascaró^2+^, Remco Folkertsma^2^, and Alice M. I. Auersperg^2^,**

**Methods**

**Pre-test procedure**

Subjects were given pre-test training to calculate the maximum weight they were willing to carry. To do this, we produced 32 identical objects (for dimensions see Figure 2), differing in mass in 2.5g increments from 10g to 90g and tested the subjects’ willingness to transport each object by flight. An object was considered ‘not carriable’ if the subject dropped, threw, or refused to carry the object to the deposit platform twice consecutively. This enabled us to find the maximum mass that each bird was willing to carry in flight. We then used this value to find a ‘comfortable’ heavy mass for each bird which was calculated as a proportion of 80% of the previously ascertained maximum weight for each bird. This proportion was chosen to prevent injury.

To ensure comparable perceived differences between the heavy and light weights across subjects, the light object had a mass of 25% of the heavy object for each bird.

**Procedure training**

The birds were given a single session of 6 trials to learn the procedure. For this, one randomised object (heavy or light) was placed in the dish, and the bird was asked to transport it to the deposit tube for a reward. As the birds were rewarded for both heavy and light objects, this step helped to reduce any heavy or light weight-bias that may have remained from previous experiments (such as1).

**Object mass information**

The differences in masses carried by each bird are likely to reflect differences in motivation, strength, and body mass.

***Table S1:* Information about the objects used for each individual in this study.**

| **Name** | **Name code** | **Age** | **Sex** | **Body mass** | **Max carriable weight** | **Experiment 1 weights and**  **% of body weight** | | | | **Experiment 2 weights and**  **% of body weight** | | **First task** |
| --- | --- | --- | --- | --- | --- | --- | --- | --- | --- | --- | --- | --- |
| Figaro | FIG | 16 | Male | 328g | 90g | 72g | 22% | 18g | 5% | 60g | 18% | Walking |
| Zozo | ZOZ | 13 | Male | 307g | 90g | 72g | 23% | 18g | 6% | 60g | 19% | Walking |
| Renki | REN | 3 | Male | 319g | 80g | 64g | 20% | 16g | 5% | 30g | 9% | Walking |
| Fini | FIN | 16 | Female | 246g | 62.5g | 50g | 20% | 12.5g | 5% | 30g | 12% | Walking |
| Titus | TIT | 6 | Male | 310g | 90g | 72g | 23% | 18g | 6% | 60g | 19% | Flying |
| Kiwi | KIW | 13 | Male | 327g | 90g | 72g | 22% | 18g | 6% | 60g | 18% | Flying |
| Dolittle | DOL | 12 | Male | 272g | 50g | 40g | 14% | 10g | 4% | 30g | 11% | Flying |
| Jane | JAN | 6 | Female | 280g | 50g | 40g | 14% | 10g | 4% | 30g | 10% | Flying |

**Individual level switching and side bias analyses**

Further analyses were conducted to understand whether differences in decision-making could be explained using side biases and switching frequencies. However, by conducting individual analyses, we vastly reduce the sample size for our tests and therefore advise caution when interpreting the results.

We conducted a Wilcoxon rank-sum test on the average switching frequency for each individual and found that though Figaro and Jane did show reduced switching compared to the other birds in the Overall weight experiment, this result was non-significant (Wilcoxon rank-sum, p-value = 0.07143).

We also investigated side biases by comparing the number of trials in which individual chose the object on the left vs right. We used binomial tests to determine whether the proportion of trials in which objects on the left were chosen differed significantly from 50\%. It was found that Figaro and Jane both showed significant side preferences (binomial test: n = 180, p-value < 0.05).


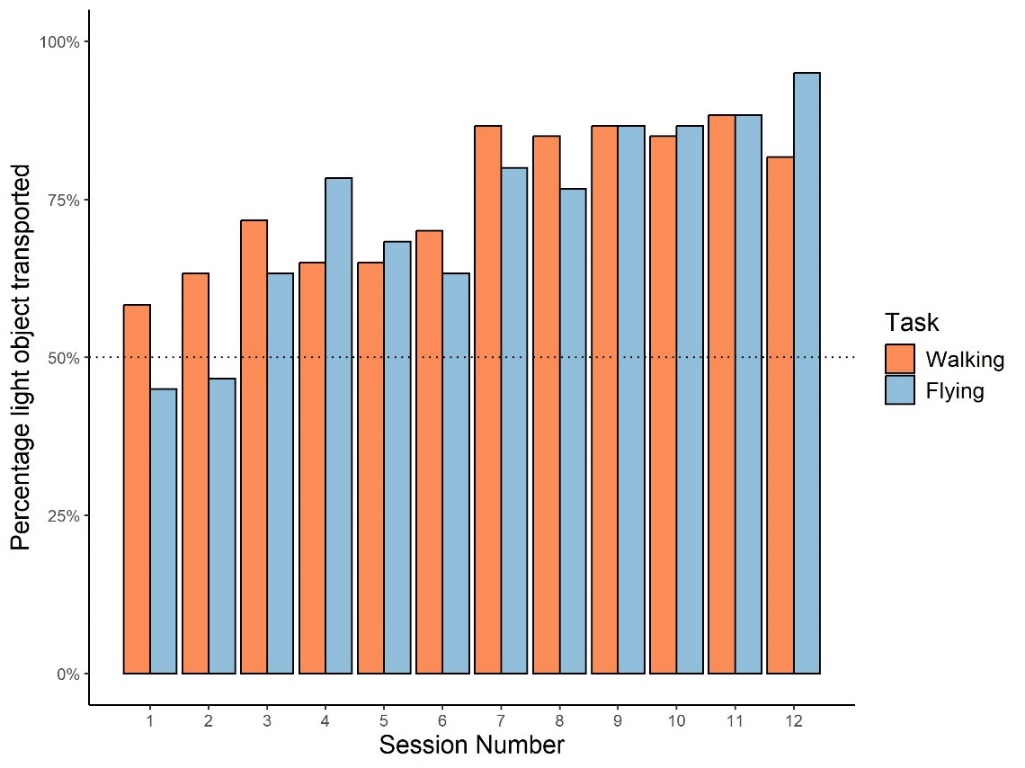


***Figure S1:*** This plot shows raw data for the overall weight experiment, averaged across all individuals for each session within each task. The height of the bar represents the percentage of light objects carried within each session of 15 trials, colour coded to show walking vs flying sessions. n=4 individuals for each bar.

***Figure S2:*** This plot shows the Overall Weight experiment model predictions for the probability of trials in which light objects were carried, across all individuals, colour coded for each task (walking and flying). The solid line indicates the fitted model, and shaded areas represent 95% confidence intervals derived from 1,000 bootstraps. There is no significant difference in slope between tasks.


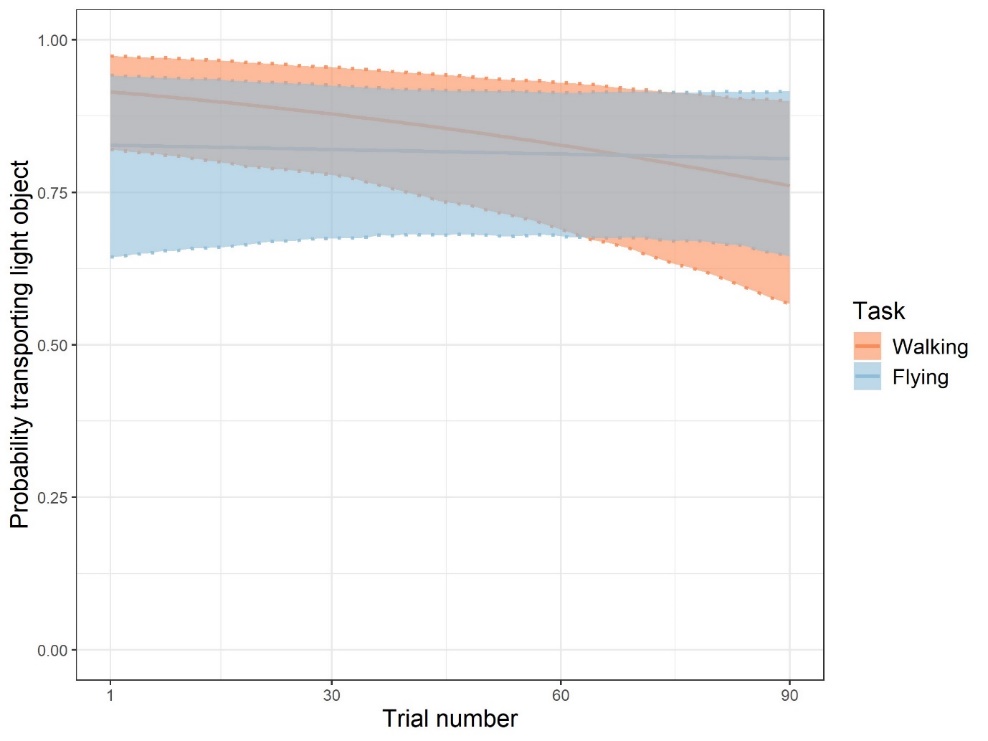


***Figure S3:*** This plot shows raw data for the weight balance experiment, averaged across all individuals for each session within each task. The height of the bar represents the percentage of balanced objects carried within each session of 15 trials, colour coded to show walking vs flying sessions. n=4 individuals for each bar.


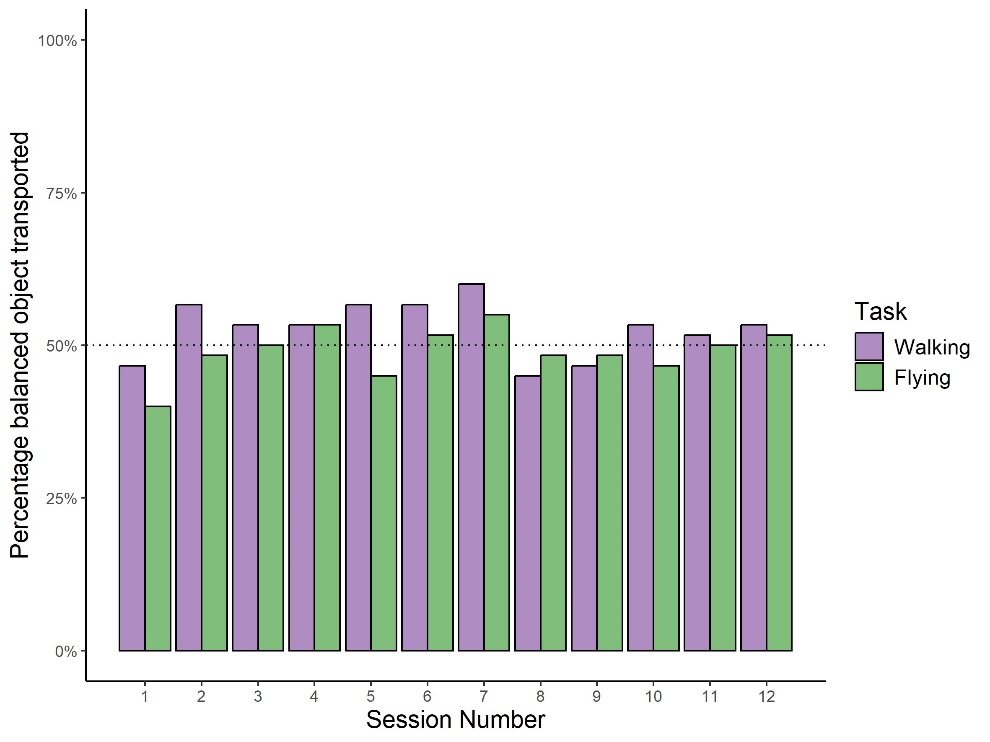

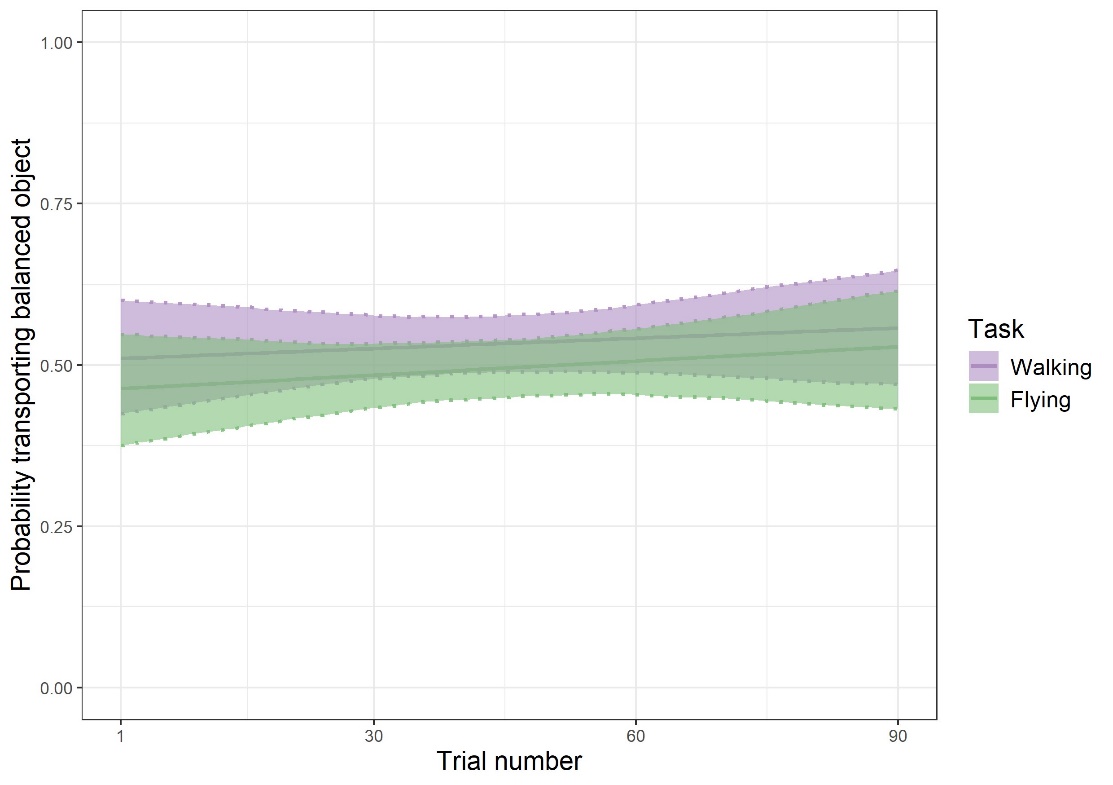


***Figure S4:*** This plot shows the Weight Balance model predictions for the probability of trials in which balanced objects were carried, across all individuals, colour coded for each task (walking and flying). The solid line indicates the fitted model, and shaded areas represent 95% confidence intervals derived from 1,000 bootstraps. There is no significant difference in slope between tasks.

***Figure S5:*** The raw data for experiment 2 (Weight Balance) showing individual variation in preferences and learning across sessions. Bars represent the percentage of light objects carried within each session of 15 trials for each individual separately, colour coded to show walking vs flying sessions.


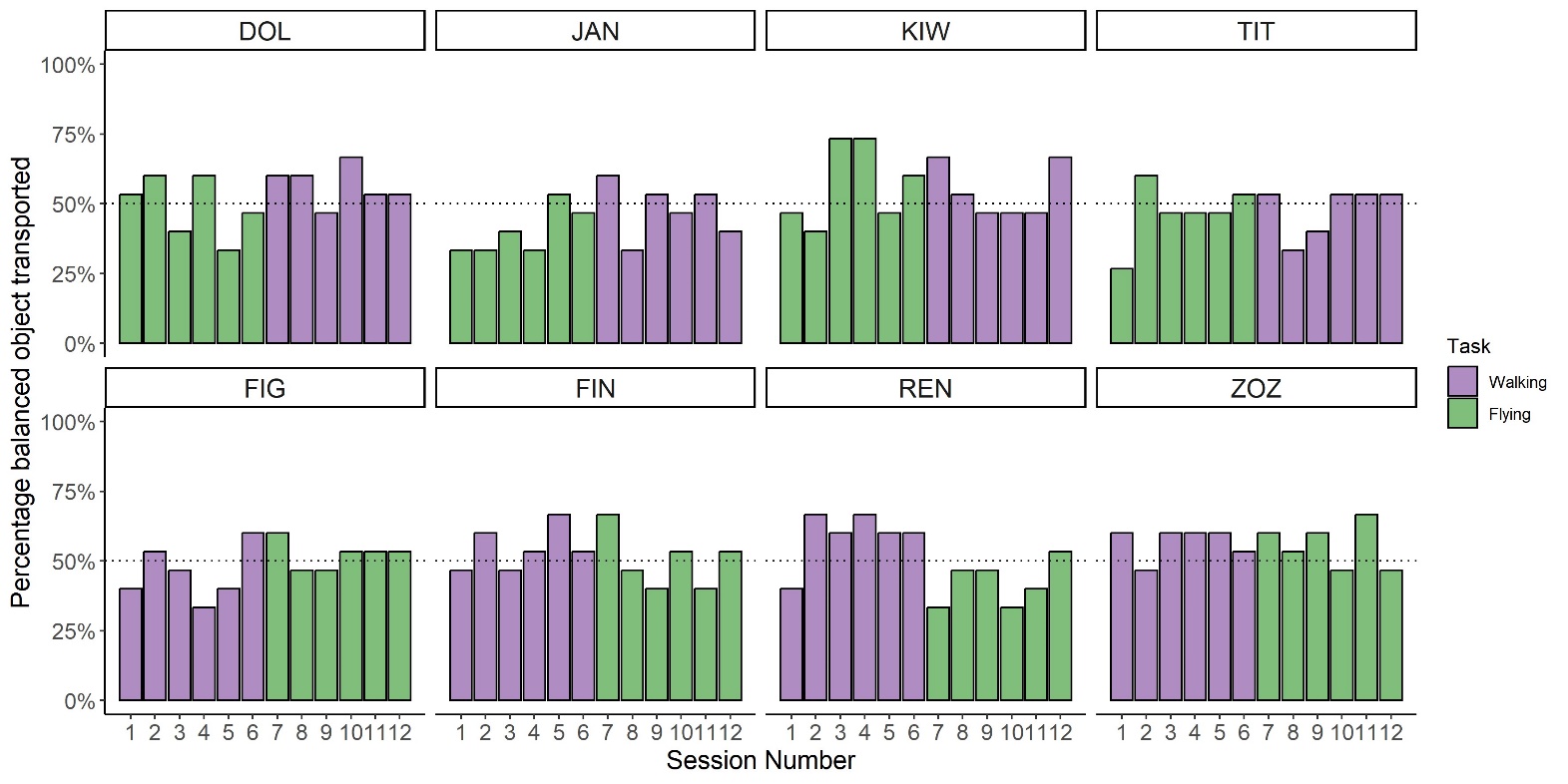

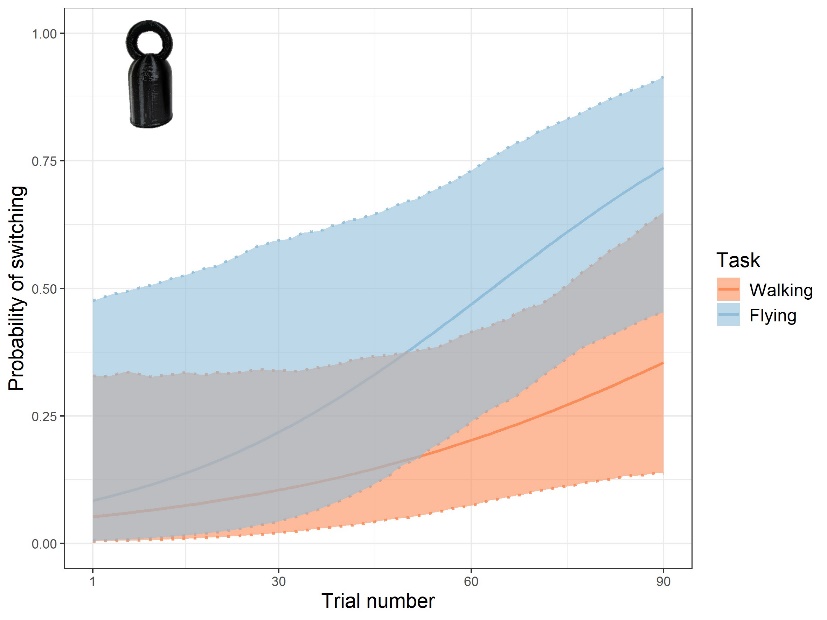

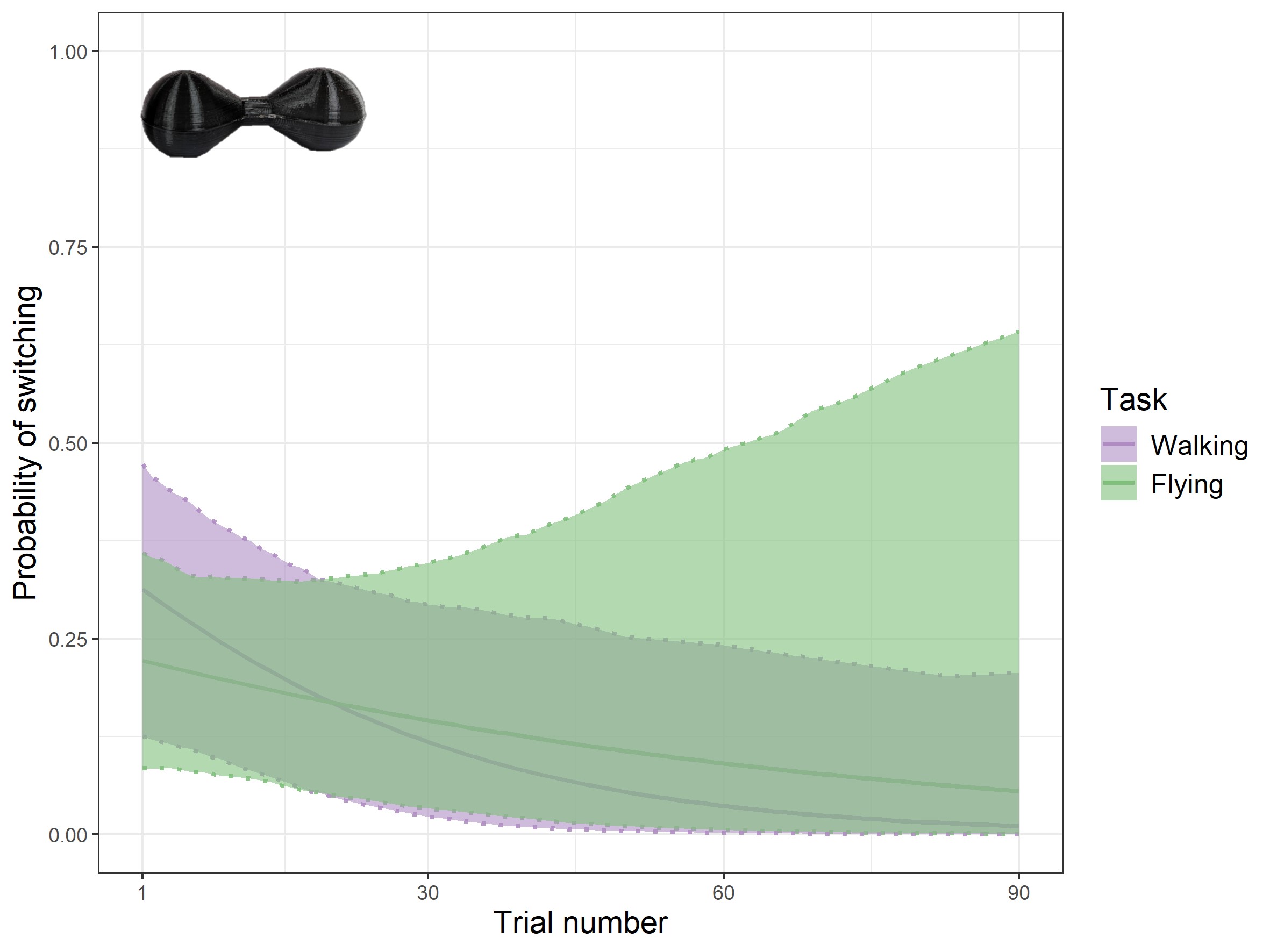


***Figure S6:*** These plots show the model estimates for switching in the first task of the overall weight experiment (plot (a)) and the weight balance experiment (plot (b)). Colours show walking vs flying tasks. For the overall weight experiment, there is not a significant difference in switching frequency between walking and flying tasks, however switching frequency does increase significantly across trials. For the weight balance experiment, there is not a significant difference in switching frequency across the walking and flying tasks but switching frequency also does not change significantly across trials.

| 1. **FULL MODEL** | | | | | | | | | |
| --- | --- | --- | --- | --- | --- | --- | --- | --- | --- |
| **Term** | **Estimate** | ***SE*** | **lower cl** | **upper cl** | ***χ*^2^** | ***df*** | ***P-value*** | **min** | **max** |
| Intercept | 1.914 | 0.454 | 1.045 | 2.941 |  |  | ^(3)^ | 1.400 | 2.334 |
| Trial number per task ^(1)^ | -0.356 | 0.147 | -0.731 | -0.058 |  |  | ^(3)^ | -0.514 | -0.233 |
| Task _(flying)_ | -0.274 | 0.303 | -1.123 | 0.382 |  |  | ^(3)^ | -0.706 | 0.076 |
| Overall trial number ^(1)^ | -0.505 | 0.190 | -0.903 | -0.112 | 5.451 | 1 | 0.020 | -0.573 | -0.404 |
| First object presented _(light)_ | 0.212 | 0.631 | -1.132 | 2.097 | 0.105 | 1 | 0.746 | -0.313 | 0.849 |
| First task _(flying)_ | 0.200 | 0.321 | -0.449 | 0.838 | 0.308 | 1 | 0.579 | -0.300 | 0.491 |
| Percentage weight ^(1)^ | 1.097 | 0.240 | 0.680 | 1.745 | 10.764 | 1 | 0.001 | 0.981 | 1.308 |
| Trial number per task * Task | 0.313 | 0.247 | -0.192 | 0.841 | 1.371 | 1 | 0.242 | 0.220 | 0.524 |
| ^(1)^ Trial number per task, percentage weight, and overall trial number, were z-transformed to a mean of 0 and a standard deviation of 1  ^(2)^ Task; first task and first object presented were dummy coded with: walking; walking; and heavy as respective reference categories  ^(3)^ Not indicated because of having a very limited interpretation | | | | | | | | | |

| 1. **REDUCED MODEL** | | | | | | | | | |
| --- | --- | --- | --- | --- | --- | --- | --- | --- | --- |
| **Term** | **Estimate** | ***SE*** | **lower cl** | **upper cl** | ***χ*^2^** | ***df*** | ***P-value*** | **min** | **max** |
| Intercept | 2.109 | 0.440 | 1.252 | 3.099 |  |  | ^(3)^ | 1.657 | 2.422 |
| Trial number per task ^(1)^ | -0.231 | 0.117 | -0.503 | -0.033 | 2.864 | 1 | 0.091 | -0.340 | 0.025 |
| Task _(flying)_ | -0.221 | 0.330 | -1.065 | 0.495 | 0.432 | 1 | 0.511 | -0.674 | 0.107 |
| First object presented _(light)_ | -0.595 | 0.195 | -0.980 | -0.230 | 6.889 | 1 | 0.009 | -0.684 | -0.469 |
| First task _(flying)_ | 0.132 | 0.622 | -1.259 | 1.769 | 0.044 | 1 | 0.835 | -0.341 | 0.726 |
| Percentage weight ^(1)^ | 0.207 | 0.310 | -0.416 | 0.825 | 0.368 | 1 | 0.544 | -0.329 | 0.445 |
| Overall trial number ^(1)^ | 1.044 | 0.255 | 0.561 | 1.698 | 9.376 | 1 | 0.002 | 0.860 | 1.329 |
| ^(1)^ Trial number per task, percentage weight, and overall trial number, were z-transformed to a mean of 0 and a standard deviation of 1  ^(2)^ Task; first task and first object presented were dummy coded with: walking; walking; and heavy as respective reference categories  ^(3)^ Not indicated because of having a very limited interpretation  ***Table S2:* Results of the full (a) and reduced (b) model of object choice for the Overall Weight experiment (1), related to Figure 1a** Estimates, together with standard errors, confidence limits, significance tests as well as minimum and maximum of estimates obtained after dropping levels of the random effects one at a time (model stability). | | | | | | | | | |

| **FULL MODEL** | | | | | | | | | |
| --- | --- | --- | --- | --- | --- | --- | --- | --- | --- |
| Term | Estimate | *SE* | lower cl | upper cl | *χ*^2^ | *df* | *P-value* | min | max |
| Intercept | -2.206 | 0.728 | -3.912 | -0.806 |  |  | ^(3)^ | -2.651 | -1.337 |
| Trial number per task ^(1)^ | 0.671 | 0.353 | -0.003 | 1.594 |  |  | ^(3)^ | 0.260 | 0.828 |
| Task _(flying)_ | 1.066 | 0.751 | -0.553 | 3.017 |  |  | ^(3)^ | -0.654 | 1.532 |
| First object presented _(light)_ | 0.322 | 0.281 | -0.227 | 0.924 | 1.350 | 1 | 0.245 | 0.131 | 0.441 |
| First side pickup _(Right)_ | 0.828 | 0.428 | -0.030 | 1.848 | 3.271 | 1 | 0.071 | 0.547 | 0.987 |
| Percentage weight ^(1)^ | 1.220 | 0.447 | 0.524 | 2.112 | 2.413 | 1 | 0.120 | -0.458 | 1.440 |
| Trial number per task * Task | 0.327 | 0.484 | -0.810 | 1.464 | 0.417 | 1 | 0.518 | -0.024 | 0.701 |
| ^(1)^ Trial number per task and percentage weight were z-transformed to a mean of 0 and a standard deviation of 1  ^(2)^ Task; first object presented; and first side pick up were dummy coded with: walking; heavy; and left as respective reference categories  ^(3)^ Not indicated because of having a very limited interpretation | | | | | | | | | |

| **REDUCED MODEL** | | | | | | | | | |
| --- | --- | --- | --- | --- | --- | --- | --- | --- | --- |
| Term | Estimate | *SE* | lower cl | upper cl | *χ*^2^ | *df* | *P-value* | min | max |
| Intercept | -2.342 | 0.725 | -3.983 | -1.030 |  |  | ^(3)^ | -2.756 | -1.056 |
| Trial number per task ^(1)^ | 0.824 | 0.289 | 0.320 | 1.490 | 5.608 | 1 | 0.018 | 0.460 | 0.972 |
| Task _(flying)_ | 1.301 | 0.783 | -0.351 | 3.054 | 1.740 | 1 | 0.187 | -1.239 | 1.710 |
| First object presented _(light)_ | 0.328 | 0.282 | -0.244 | 0.926 | 1.397 | 1 | 0.237 | 0.109 | 0.443 |
| First side pickup _(Right)_ | 0.832 | 0.417 | -0.067 | 1.788 | 3.426 | 1 | 0.064 | 0.565 | 1.048 |
| Percentage weight ^(1)^ | 1.238 | 0.491 | 0.496 | 2.155 | 2.312 | 1 | 0.128 | -0.370 | 1.452 |
| ^(1)^ Trial number per task and percentage weight were z-transformed to a mean of 0 and a standard deviation of 1  ^(2)^ Task; first object presented; and first side pick up were dummy coded with: walking; heavy; and left as respective reference categories  ^(3)^ Not indicated because of having a very limited interpretation  ***Table S3:* Results of the full (a) and reduced (b) model of switching for the Overall Weight experiment (1), related to Figure S6a** Estimates, together with standard errors, confidence limits, significance tests as well as minimum and maximum of estimates obtained after dropping levels of the random effects one at a time (model stability). | | | | | | | | | |

| **FULL MODEL** | | | | | | | | | |
| --- | --- | --- | --- | --- | --- | --- | --- | --- | --- |
| Term | Estimate | *SE* | lower cl | upper cl | *χ*^2^ | *df* | *P-value* | min | max |
| Intercept | -1.767 | 0.470 | -2.806 | -0.867 |  |  | ^(3)^ | -2.078 | -1.193 |
| Trial number per task ^(1)^ | 0.563 | 0.243 | 0.110 | 1.142 |  |  | ^(3)^ | 0.439 | 0.782 |
| Task _(flying)_ | 0.523 | 0.646 | -0.831 | 1.855 |  |  | ^(3)^ | -0.040 | 1.227 |
| First object presented _(light)_ | 0.019 | 0.163 | -0.299 | 0.333 | 0.014 | 1 | 0.907 | -0.052 | 0.089 |
| First side pickup _(Right)_ | 0.445 | 0.239 | -0.069 | 0.947 | 3.126 | 1 | 0.077 | 0.270 | 0.582 |
| Percentage weight ^(1)^ | 0.613 | 0.313 | 0.063 | 1.167 | 3.061 | 1 | 0.080 | 0.291 | 0.948 |
| Trial number per task * Task | 0.027 | 0.333 | -0.767 | 0.740 | 0.000 | 1 | 1.000 | -0.164 | 0.205 |
| ^(1)^ Trial number per task and percentage weight were z-transformed to a mean of 0 and a standard deviation of 1  ^(2)^ Task; first object presented; and first side pick up were dummy coded with: walking; heavy; and left as respective reference categories  ^(3)^ Not indicated because of having a very limited interpretation | | | | | | | | | |

| **REDUCED MODEL** | | | | | | | | | |
| --- | --- | --- | --- | --- | --- | --- | --- | --- | --- |
| Term | Estimate | *SE* | lower cl | upper cl | *χ*^2^ | *df* | *P-value* | min | max |
| Intercept | -1.788 | 0.416 | -2.630 | -0.954 |  |  | ^(3)^ | -2.022 | -1.248 |
| Trial number per task ^(1)^ | 0.576 | 0.181 | 0.240 | 0.925 | 7.616 | 1 | 0.006 | 0.421 | 0.687 |
| Task _(flying)_ | 0.559 | 0.494 | -0.485 | 1.617 | 1.296 | 1 | 0.255 | 0.050 | 1.084 |
| First object presented _(light)_ | 0.019 | 0.162 | -0.336 | 0.355 | 0.013 | 1 | 0.910 | -0.052 | 0.087 |
| First side pickup _(Right)_ | 0.443 | 0.238 | -0.064 | 0.912 | 3.151 | 1 | 0.076 | 0.260 | 0.579 |
| Percentage weight ^(1)^ | 0.618 | 0.314 | 0.106 | 1.134 | 3.103 | 1 | 0.078 | 0.300 | 0.938 |
| ^(1)^ Trial number per task and percentage weight were z-transformed to a mean of 0 and a standard deviation of 1  ^(2)^ Task; first object presented; and first side pick up were dummy coded with: walking; heavy; and left as respective reference categories  ^(3)^ Not indicated because of having a very limited interpretation | | | | | | | | |  |
|  |  |  |  |  |  |  |  |  |  |
|  |  |  |  |  |  |  |  |  |  |

***Table S4:* Results of the full (a) and reduced (b) model of number of switches for the Overall Weight experiment (1).** Estimates, together with standard errors, confidence limits, significance tests as well as minimum and maximum of estimates obtained after dropping levels of the random effects one at a time (model stability).

| **FULL MODEL** | | | | | | | | | | | | | | | | | |  |
| --- | --- | --- | --- | --- | --- | --- | --- | --- | --- | --- | --- | --- | --- | --- | --- | --- | --- | --- |
| Term | | Estimate | | *SE* | | lower cl | | upper cl | | *χ*^2^ | *df* | | *P-value* | | min | | max |  |
| Intercept | | -0.113 | | 0.387 | | -0.899 | | 0.692 | |  |  | | ^(3)^ | | -0.386 | | 0.169 |  |
| Trial number per task ^(1)^ | | 0.056 | | 0.093 | | -0.118 | | 0.241 | |  |  | | ^(3)^ | | 0.002 | | 0.100 |  |
| Task _(flying)_ | | -0.153 | | 0.123 | | -0.369 | | 0.101 | |  |  | | ^(3)^ | | -0.239 | | -0.042 |  |
| Overall trial number ^(1)^ | | -0.005 | | 0.071 | | -0.144 | | 0.133 | | 0.004 | 1 | | 0.949 | | -0.054 | | 0.059 |  |
| First object presented _(not balanced)_ | | 0.600 | | 0.745 | | -0.942 | | 2.058 | | 0.624 | 1 | | 0.430 | | 0.265 | | 1.110 |  |
| First task _(flying)_ | | -0.104 | | 0.126 | | -0.374 | | 0.137 | | 0.627 | 1 | | 0.428 | | -0.195 | | 0.007 |  |
| Percentage weight ^(1)^ | | 0.056 | | 0.063 | | -0.060 | | 0.175 | | 0.707 | 1 | | 0.401 | | -0.001 | | 0.096 |  |
| Trial number per task * Task | | 0.021 | | 0.122 | | -0.213 | | 0.265 | | 0.027 | 1 | | 0.869 | | -0.037 | | 0.072 |  |
| ^(1)^ Trial number per task, percentage weight, and overall trial number, were z-transformed to a mean of 0 and a standard deviation of 1  ^(2)^ Task; first object presented; and first task were dummy coded with: walking; balanced; and walking as respective reference categories  ^(3)^ Not indicated because of having a very limited interpretation | | | | | | | | | | | | | | | | | |  |
| **REDUCED MODEL** | | | | | | | | | | | | | | | | | | |
| Term | Estimate | | *SE* | | lower cl | | upper cl | | *χ*^2^ | | | *df* | | *P-value* | | min | max | |
| Intercept | -0.112 | | 0.387 | | -0.888 | | 0.641 | |  | | |  | | ^(3)^ | | -0.384 | 0.170 | |
| Trial number per task ^(1)^ | 0.066 | | 0.071 | | -0.083 | | 0.200 | | 0.834 | | | 1 | | 0.361 | | 0.017 | 0.100 | |
| Task _(flying)_ | -0.155 | | 0.123 | | -0.391 | | 0.096 | | 1.486 | | | 1 | | 0.223 | | -0.240 | -0.044 | |
| First object presented _(light)_ | 0.601 | | 0.745 | | -0.898 | | 2.105 | | 0.625 | | | 1 | | 0.429 | | 0.265 | 1.110 | |
| First task _(flying)_ | -0.104 | | 0.126 | | -0.349 | | 0.144 | | 0.633 | | | 1 | | 0.426 | | -0.195 | 0.008 | |
| Percentage weight ^(1)^ | 0.056 | | 0.063 | | -0.070 | | 0.182 | | 0.711 | | | 1 | | 0.399 | | -0.001 | 0.096 | |
| Overall trial number ^(1)^ | -0.004 | | 0.071 | | -0.138 | | 0.123 | | 0.004 | | | 1 | | 0.952 | | -0.054 | 0.059 | |
| ^(1)^ Trial number per task, percentage weight, and overall trial number, were z-transformed to a mean of 0 and a standard deviation of 1  ^(2)^ Task; first object presented; and first task were dummy coded with: walking; balanced; and walking as respective reference categories  ^(3)^ Not indicated because of having a very limited interpretation | | | | | | | | | | | | | | | | | | |

***Table S5:* Results of the full (a) and reduced (b) model of object choice for the Weight Balance experiment (2), related to Figure 1b** Estimates, together with standard errors, confidence limits, significance tests as well as minimum and maximum of estimates obtained after dropping levels of the random effects one at a time (model stability).

| **FULL MODEL** | | | | | | | | | |
| --- | --- | --- | --- | --- | --- | --- | --- | --- | --- |
| **Term** | **Estimate** | ***SE*** | **lower cl** | **upper cl** | ***χ*^2^** | ***df*** | ***P-value*** | **min** | **max** |
| Intercept | -3.316 | 0.931 | -5.886 | -1.588 |  |  | ^(3)^ | -2.718 | -2.718 |
| Trial number per task ^(1)^ | -1.097 | 0.534 | -2.506 | -0.021 |  |  | ^(3)^ | -0.565 | -0.565 |
| Task _(flying)_ | 0.618 | 1.296 | -2.039 | 3.542 |  |  | ^(3)^ | 3.128 | 3.128 |
| First object presented _(light)_ | 0.014 | 0.283 | -0.596 | 0.636 | 0.245 | 1 | 0.621 | 0.097 | 0.097 |
| First side pickup _(F)_ | 1.132 | 0.586 | 0.000 | 2.717 | 0.692 | 1 | 0.406 | 1.637 | 1.637 |
| Percentage weight ^(1)^ | 0.143 | 0.281 | -0.492 | 0.795 | 0.245 | 1 | 0.621 | 0.651 | 0.651 |
| Trial number per task * Task | 0.634 | 0.730 | -0.976 | 2.327 | 0.692 | 1 | 0.406 | 1.584 | 1.584 |
| ^(1)^ Trial number per task and percentage weight were z-transformed to a mean of 0 and a standard deviation of 1  ^(2)^ Task; first object presented; and first side pick up were dummy coded with: walking; heavy; and B as respective reference categories  ^(3)^ Not indicated because of having a very limited interpretation | | | | | | | | | |
| **REDUCED MODEL** | | | | | | | | | |
| **Term** | **Estimate** | ***SE*** | **lower cl** | **upper cl** | ***χ*^2^** | ***df*** | ***P-value*** | **min** | **max** |
| Intercept | -2.830 | 0.759 | -5.153 | -1.431 |  |  | ^(3)^ | -3.502 | -2.398 |
| Trial number per task ^(1)^ | -0.788 | 0.371 | -1.656 | -0.064 | 4.844 | 1 | 0.028 | -1.001 | -0.495 |
| Task _(flying)_ | -0.416 | 0.569 | -1.839 | 0.800 | 0.751 | 1 | 0.386 | -0.991 | 0.328 |
| First object presented _(light)_ | -0.012 | 0.277 | -0.611 | 0.580 | 0.002 | 1 | 0.965 | -0.128 | 0.075 |
| First side pickup _(F)_ | 1.172 | 0.594 | 0.112 | 2.869 | 3.661 | 1 | 0.056 | 0.745 | 1.634 |
| Percentage weight ^(1)^ | 0.142 | 0.277 | -0.477 | 0.796 | 0.245 | 1 | 0.621 | -0.506 | 0.411 |
| ^(1)^ Trial number per task and percentage weight were z-transformed to a mean of 0 and a standard deviation of 1  ^(2)^ Task; first object presented; and first side pick up were dummy coded with: walking; heavy; and B as respective reference categories  ^(3)^ Not indicated because of having a very limited interpretation  ***Table S6:* Results of the full (a) and reduced (b) model of switching for the Weight Balance experiment (2), related to Figure S6b** Estimates, together with standard errors, confidence limits, significance tests as well as minimum and maximum of estimates obtained after dropping levels of the random effects one at a time (model stability). | | | | | | | | | |

| **FULL MODEL** | | | | | | | | | | | | | | | | | |
| --- | --- | --- | --- | --- | --- | --- | --- | --- | --- | --- | --- | --- | --- | --- | --- | --- | --- |
| **Term** | **Estimate** | ***SE*** | | **lower cl** | | **upper cl** | | ***χ*^2^** | | ***df*** | ***P-value*** | | **min** | | **max** | | |
| Intercept | -2.483 | 0.708 | | -3.951 | | -1.121 | |  | |  | ^(3)^ | | -3.083 | | -2.149 | | |
| Trial number per task ^(1)^ | -0.801 | 0.374 | | -1.572 | | -0.072 | |  | |  | ^(3)^ | | -1.069 | | -0.544 | | |
| Task _(flying)_ | -0.011 | 0.928 | | -1.840 | | 1.747 | |  | |  | ^(3)^ | | -1.583 | | 0.659 | | |
| First object presented _(light)_ | -0.086 | 0.161 | | -0.472 | | 0.251 | | 0.274 | | 1 | 0.601 | | -0.172 | | -0.007 | | |
| First side pickup _(F)_ | 0.547 | 0.362 | | -0.203 | | 1.446 | | 2.128 | | 1 | 0.145 | | 0.295 | | 0.776 | | |
| Percentage weight ^(1)^ | -0.350 | 0.460 | | -1.274 | | 0.558 | | 0.563 | | 1 | 0.453 | | -0.710 | | -0.068 | | |
| Trial number per task * Task | 0.235 | 0.521 | | -0.748 | | 1.292 | | 0.184 | | 1 | 0.668 | | -0.509 | | 0.599 | | |
| ^(1)^ Trial number per task and percentage weight were z-transformed to a mean of 0 and a standard deviation of 1 | | | | | | | | | | | |  | |  | |  |  |
| ^(2)^ Task; first object presented; and first side pick up were dummy coded with: walking; heavy; and B as respective reference categories | | | | | | | | | | | | | |  | |  |  |
| ^(3)^ Not indicated because of having a very limited interpretation | | |  | |  | |  | |  | | |  | |  | |  |  |

| **REDUCED MODEL** | | | | | | | | | | |
| --- | --- | --- | --- | --- | --- | --- | --- | --- | --- | --- |
| **Term** | **Estimate** | ***SE*** | **lower cl** | **upper cl** | ***χ*^2^** | ***df*** | ***P-value*** | **min** | **max** | |
| Intercept | -2.473 | 0.716 | -4.034 | -1.055 |  |  | ^(3)^ | -3.031 | -2.141 | |
| Trial number per task ^(1)^ | -0.687 | 0.274 | -1.289 | -0.162 | 5.458 | 1 | 0.019 | -0.962 | -0.513 | |
| Task _(flying)_ | -0.058 | 0.934 | -1.927 | 1.755 | 0.004 | 1 | 0.951 | -1.283 | 0.614 | |
| First object presented _(light)_ | -0.085 | 0.162 | -0.416 | 0.277 | 0.270 | 1 | 0.603 | -0.150 | -0.006 | |
| First side pickup _(F)_ | 0.552 | 0.373 | -0.250 | 1.356 | 2.051 | 1 | 0.152 | 0.286 | 0.764 | |
| Percentage weight ^(1)^ | -0.350 | 0.466 | -1.261 | 0.562 | 0.549 | 1 | 0.459 | -0.793 | -0.079 | |
| ^(1)^ Trial number per task and percentage weight were z-transformed to a mean of 0 and a standard deviation of 1  ^(2)^ Task; first object presented; and first side pick up were dummy coded with: walking; heavy; and B as respective reference categories  ^(3)^ Not indicated because of having a very limited interpretation | | | | | | | | | |  |

***Table S7:* Results of the full (a) and reduced (b) model of number of switches for the Weight Balance experiment (2).** Estimates, together with standard errors, confidence limits, significance tests as well as minimum and maximum of estimates obtained after dropping levels of the random effects one at a time (model stability).

| **subject.ID** | **heavy.object transported** | **light.object transported** | **% light** | **p-value** |
| --- | --- | --- | --- | --- |
| DOL | 31 | 149 | 0.827778 | < 0.001 |
| FIG | 79 | 101 | 0.561111 | 0.1173 |
| FIN | 62 | 118 | 0.655556 | < 0.001 |
| JAN | 84 | 96 | 0.533333 | 0.4124 |
| KIW | 50 | 130 | 0.722222 | < 0.001 |
| REN | 23 | 157 | 0.872222 | < 0.001 |
| TIT | 28 | 152 | 0.844444 | < 0.001 |
| ZOZ | 12 | 168 | 0.933333 | < 0.001 |

***Table S8:* Results of the binomial test for overall experimental object choice (across all trials, irrespective of task) in the Overall Weight experiment.**

| **subject.ID** | **Left** | **Right** | **% Left** | ***P-*value** |
| --- | --- | --- | --- | --- |
| FIG | 163 | 17 | 0.905556 | 2.2e-16 |
| JAN | 49 | 131 | 0.272222 | 8.018e-10 |

***Table S9:* Results of the binomial test for side bias in birds showing no significant preference in the Overall Weight experiment (1).**

|  | **Number of switches per trial per individual** | | | |
| --- | --- | --- | --- | --- |
|  | **Overall Weight (1)** | | **Weight Balance (2)** | |
| **subject.ID** | **Average** | **Maximum** | **Average** | **Maximum** |
| DOL | 0.494444 | 4 | 0.172222 | 6 |
| FIG | 0.116667 | 3 | 0.05 | 1 |
| FIN | 1.216667 | 13 | 0.611111 | 16 |
| JAN | 0.077778 | 1 | 0.05 | 2 |
| KIW | 0.977778 | 8 | 0.655556 | 7 |
| REN | 0.955556 | 6 | 0.472222 | 6 |
| TIT | 0.388889 | 1 | 0.105556 | 5 |
| ZOZ | 0.85 | 6 | 0.122222 | 4 |

***Table S10:* Further investigation of the average and maximum number of switches within each trial per individual in Overall weight (1) and Weight balance (2) experiments.**

***S1 Video caption:***

Video showing the apparatus, objects, and workflow of the experiment. Video shows one bird completing parts of the task to show the progress of a single trial. These videos were produced after the experiment was completed by all birds. The videos were taken outside of the experimental testing room where experiments actually took place.

0:00 – 0:05 – Cover slide with name of study, contributing authors, and journal of submission

0:05 – 0:16 – Side and front views of the apparatus and objects for walking trials

0:17 – 0:33 – Video of bird completing a walking trial

0:34 – 0:46 – Side and front views of the apparatus and objects for flying trials #

0:47 – 0:51 – Video of bird switching

0:52 – 0:59 – Slow motion of bird completing flying trial

1:00 – 1:06 – Cover slide with name of study, contributing authors, and journal of submission
